# Virtual Morality: Transitioning from Moral Judgment to Moral Action?

**DOI:** 10.1371/journal.pone.0164374

**Published:** 2016-10-10

**Authors:** Kathryn B. Francis, Charles Howard, Ian S. Howard, Michaela Gummerum, Giorgio Ganis, Grace Anderson, Sylvia Terbeck

**Affiliations:** 1 University of Plymouth, School of Psychology, Drake Circus, Plymouth, PL1 4AA, United Kingdom; 2 University of Plymouth, Centre for Robotics and Neural Systems, Drake Circus, Plymouth, PL1 4AA, United Kingdom; Tsinghua University, CHINA

## Abstract

The nature of moral action versus moral judgment has been extensively debated in numerous disciplines. We introduce Virtual Reality (VR) moral paradigms examining the action individuals take in a high emotionally arousing, direct action-focused, moral scenario. In two studies involving qualitatively different populations, we found a greater endorsement of utilitarian responses–killing one in order to save many others–when action was required in moral virtual dilemmas compared to their judgment counterparts. Heart rate in virtual moral dilemmas was significantly increased when compared to both judgment counterparts and control virtual tasks. Our research suggests that moral action may be viewed as an independent construct to moral judgment, with VR methods delivering new prospects for investigating and assessing moral behaviour.

## Introduction

Life is full of examples of not “practicing what you preach” (e.g., [1]). Consider Shakespeare’s Iago as the classic hypocrite; a modest character in public but an immoral character when alone declaring that “I am not what I am” ([2], p. 7). Numerous examples of moral inconsistencies can be found for individuals who demonstrate a disparity between how they say they will act and how they actually act [3]. Despite the abundance of these real-life moral inconsistencies, the relationship between moral judgment and action remains unclear [4].

People’s moral judgments have been investigated using hypothetical moral dilemmas borrowed from philosophy (e.g., [5]). The classic example is the “trolley problem” comprising both the “footbridge” and “switch” dilemmas [6]. In the footbridge dilemma, when faced with the prospect of pushing a large man off a bridge in order to stop an approaching trolley threatening to kill five construction workers, the majority of people say that they would disapprove of this harmful action. However, in the switch dilemma, when faced with the task of switching a trolley’s direction to kill one worker instead of five, the majority of people will approve [5].

### Dual-Process Models of Moral Judgment

In order to understand these differing responses to the footbridge and switch dilemmas, various dual-system frameworks have been proposed [5, 7]. According to Greene, Sommerville [5], moral judgments are driven by distinct and sometimes competing processes. First, intuitive emotional processing is thought to relate to “deontological” or non-utilitarian judgment, seeking to promote rights and duties (i.e., refusing to endorse harmful actions). Second, controlled cognitive processing is thought to relate to utilitarian judgment, seeking to maximise welfare for the largest number of people. In Greene’s model, these responses are affected by the type of dilemma. “Personal” dilemmas are those that cause another person(s) harm and this is not “a deflection of an existing threat onto a different party” ([8], p. 389). Dilemmas not meeting these criteria are termed “impersonal”. Thus personal dilemmas, such as the footbridge dilemma, are thought to trigger increased activity in brain areas associated with emotional processing resulting in an immediate negative emotional response. In impersonal dilemmas, such as the switch dilemma, an absence of this conflicting emotional response results in increased activations in areas involved in working memory and could mean that individuals revert to utilitarian mode [5]. In terms of the dualism proposed in Greene’s model, research suggests that the arbitrary division of cognition and emotion can be somewhat artificial [8] and is far from straightforward.

In an attempt to remove this distinction, Cushman [7] proposed a second dual-process framework in his action, outcome and value model. Concepts of model-based and model-free reinforcement learning are incorporated to offer insights into moral decision making. This model distinguishes between a process that assigns value to actions (e.g., pushing the man off the bridge) and a process that assigns value to the outcome instead (e.g., causing the man severe harm). Cushman argues that both processes involve emotional and cognitive elements and are not mutually exclusive. The switch dilemma is processed by a model-based system which favours saving lives (outcome-based value). Whereas the footbridge dilemma is more complex, involving a model-free system which assigns negative value to the act of pushing (action-based value) as this typically leads to negative outcomes.

### Moral Actions in “Virtual Reality”

Whilst there is strong evidence to support dual-system frameworks of moral judgment [5, 9–11], little research has attempted to understand the relationship between theoretical moral judgments and moral actions [12]. Does refusing to push the man off the footbridge reflect a theoretical or normative decision, or does it reflect a behavioural decision; “would someone … actually resort to this course of action when the full repertoire of contextual features comes into play?” ([12], p. 95).

Attempting to utilise Greene’s dual process model [5] in the framework of moral action, Navarrete, McDonald [13] created the switch dilemma using Virtual Reality (VR). Virtual actions were compared to hypothetical moral judgments and electrodermal activity was measured to assess arousal. In both the action and judgment conditions, the majority of people endorsed utilitarian outcomes. Increased emotional arousal was found to be associated with a decrease in utilitarian endorsements. This supports the theory that activations of emotional systems are associated with non-utilitarian moral judgments [5]. This, along with identical outcomes of a further virtual study, supports the generalisation of Greene’s model to the context of moral action [5, 14]. In contrast, Patil, Cogoni [12] found that utilitarian responses were greater for impersonal dilemmas in VR than for their judgment counterparts. They argue that Cushman’s model explains their findings; the saliency of the virtual environment meant that the outcome-based value associated with not acting to save endangered victims had a stronger negative value, than choosing to carry out a harmful action against one individual [12]. Although this VR research has provided the foundations for studying moral action in arousing impersonal dilemmas, the incorporation of a personal dilemma might offer insights into decision-making in emotionally conflicting situations [12, 13]. The current study aims to do just that.

### Moral Action as Distinct from Moral Judgment

Morality and action possibility have been linked in adaptive frameworks examining perceptions of morality and how this subsequently regulates social behaviours [15]. The predictive element of morality is thought to play a subsequent role in social perception in aiding the identification of potentially beneficial or harmful group members [16]. For example, individual’s expressing non-utilitarian values are perceived as more trustworthy and are subsequently preferred as partners in a social context [17]; demonstrating this adaptive function. Importantly, this predictive quality is not limited to the physical environment as Iachini, Pagliaro [18] found that moral and immoral descriptions of virtual avatars affected subsequent actions in VR. This finding that moral judgments influence behavioural regulation raises an important distinction in the present research; here we aim to examine the relationship between moral judgment and moral action rather than moral judgments and implications for action.

In this examination of the relationship between moral judgment and moral action, it must be considered that moral actions are driven by distinct mechanisms to those used for moral judgments (e.g., [19, 20]). For example, in studies examining patients with psychopathy, understanding of moral norms remains intact, which disagrees with the immoral, anti-social acts that these patients, in reality, often engage in [21, 22]. This also corresponds to the finding that healthy individuals with high psychopathic traits and low Honesty-Humility endorse utilitarian responses for action-choice questions (“Would you do it?”) but not judgment questions (“Is it morally acceptable?”) (e.g., [20, 23]). It might be that deficits in psychopathic individuals’ empathy, more specifically Empathic Concern, results in this utilitarian trend [24]. In the virtual study conducted by Navarrete, McDonald [13], the authors acknowledge that the role of personality in this virtual moral action framework should be further investigated.

### The Present Study

To our knowledge, this paper is the first to implement a personal dilemma (the footbridge dilemma) in immersive VR. Furthermore, for the first time, we also address the question of whether personality traits predict moral judgments and/or moral actions. In the present study, moral actions refer to those simulated in virtual moral dilemmas as opposed to action-choice questions which arguably remain self-reported moral judgments in nature [12].

### Study 1

Study 1 aimed to explore the relationship between theoretical moral judgments and moral actions to see whether they are associated or distinct using a personal moral dilemma. If Greene’s dual-process theory applies to moral actions (e.g., [13]), we would expect individuals to make as few utilitarian responses in VR as in hypothetical personal moral dilemmas [5]. On the other hand, if Cushman’s dual-process model applies, as described by Patil, Cogoni [12], we might expect stronger negative value to be assigned to outcomes in the virtual scenario, leading to a greater number of utilitarian responses [7].

Study 1 also aimed to assess the role of personality traits, including trait psychopathy, in predicting moral judgments and/or moral actions. Given the evidence for a dissociation between psychopaths’ responses to moral judgments and their actions in real-life [22], we theorise that traits associated with Psychopathy such as low empathy and Honesty-Humility will predict actions in VR (e.g., [20, 23]). Additionally, according to the findings of Tassy, Deruelle [20], we theorise that these traits will predict utilitarian responses for action-choice questions (“Would you do it?”) but not judgment questions (“Is it morally acceptable to?”).

In Study 1, physiological arousal was measured in the form of heart rate response. Given the novelty and visual saliency of VR, heart rate response was assessed in control tasks and also experimental (moral) tasks to primarily examine whether arousal was triggered by modality or moral context. Additionally, according to existing dual-process models of moral judgment [5] and previous virtual paradigms assessing arousal [13], we might expect an increased heart rate change to predict a non-utilitarian response.

## Method

### Participants

Forty participants comprising 35 females and 5 males, (*M*_*age*_ *=* 26.00, *SD* = 9.77 years, from 18 to 52 years) were recruited from the Plymouth University, School of Psychology, participant pool and participated for course credit. Using G* Power 3.1.9.2 with previous studies examining moral judgments as a framework [12, 25], we determined a sample size of *N* = 40, with power at 0.80 and *p* = .05, as adequate. All participants had normal or corrected-to-normal vision. The majority of participants were right-handed (92.5%). This research received ethical approval from Plymouth University Ethics Committee with written consent obtained from all individuals.

### Personality measures

All participants were asked to complete an electronic questionnaire comprising three self-report questionnaires:

The *Levenson Psychopathy Scale (LSRP)* [26] is a self-report measure of Psychopathy intended for research purposes. It has a two-factor structure assessing both primary (i.e., selfishness) (16 items; α = .81) and secondary psychopathic traits (i.e., impulsivity) (10 items; α = .68) in non-institutionalised populations. The scale contains 26 items total, rated on a 4-point Likert scale (from 1 = *strongly disagree* to 4 = *strongly agree*). The scale includes items such as “*For me*, *what’s right is whatever I can get away with*”.

The *Hexaco-IP-PR* [27] is a personality inventory designed to assess six dimensions of personality. The inventory assesses the characteristics of Honesty-Humility (Items 10; α = .82), Emotionality (Items 10; α = .85), Extraversion (Items 10; α = .67), Agreeableness (Items 10; α = .82), Conscientiousness (Items 10; α = .80) and Openness to experience (Items 10; α = .84). The inventory contains 60 items with responses given on a 5-point Likert scale (from 1 = *strongly disagree* to 5 = *strongly agree*). The inventory contains items such as “*I wouldn’t pretend to like someone just to get that person to do favours for me*”.

The *Interpersonal Reactivity Index (IRI)* [28] is an inventory designed to measure dispositional empathy. It contains four subscales to measure Perspective Taking, Empathic Concern, Personal Distress, and Fantasy. The inventory contains 28 items with responses given on a 5-point Likert scale (from A = *Does not describe me well* to E = *Describes me very well*). The scale contains items such as “*I really get involved with the feelings of characters in a novel*” (α = .80–.85).

### Moral judgment and action measures

**Fig 1:**
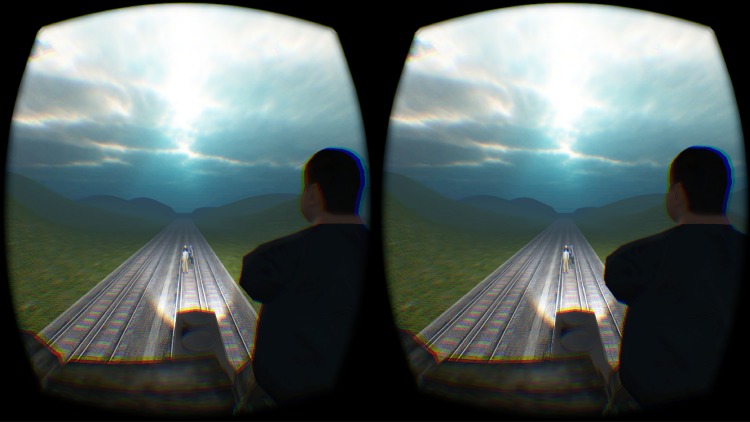
Stereoscopic image showing a scene from the footbridge virtual dilemma through Oculus Rift head-mounted display. The image is taken from the perspective of the agent at the end of the scenario in which the trolley car is about to collide with five virtual avatars standing on the tracks below. Participants are able to rotate in the virtual environment with voice commands included to ensure full understanding of the events being played out.

The study comprised two conditions to which participants were randomly allocated; a judgment condition (*N* = 20) and an action condition (*N* = 20). In the action condition, participants were first given a virtual control task that required them to push a virtual object in space after hearing a tone. This task was included to ensure that increased arousal could be attributed to the moral nature of the experimental scenario as opposed to the saliency of the virtual modality. The experimental task in the action condition was an audio-visual VR version of the footbridge dilemma as described in Foot [6]. In the scenario, the participant viewed the scene in first person view. The landscape in the virtual scenario was kept neutral with hills in the background and a neutral “skybox” or representation of the sky was incorporated. Specifically, participants stood on a footbridge with a large virtual human standing in front of them. Next, a trolley car (modern train railcar) approached from behind and travelled towards five virtual humans standing on the tracks in front of the participant (Fig 1). Participants had to decide whether they wanted to push the large person off the bridge to stop the trolley car’s progress or to allow the trolley car to continue and kill the five people standing on the tracks. Both the control task and experimental task were programmed in JavaScript within a Unity 3D game software environment. Verbal instructions played during the 3D scenario and specific instructions were given prior to the experimental task, explaining that this task involved a joystick but that participants would be given a choice about whether they wanted to interact with the virtual object or not. The VR dilemma began with a 30 second period of ambient noise and no verbal instructions to allow the participants to familiarise themselves with the virtual environment. After 30 seconds, verbal instructions informed participants that a trolley car was approaching (“*Look behind you*, *a train is coming*.”). After a further 25 seconds, a second verbal dialogue then followed (“*Hey I am too far away but if you want to save the people you could push the large person on to the tracks and derail the train*. *If you’re going to push him*, *do it now*, *but it is your choice*.”). Participants were then given a maximum of ten seconds to respond to the dilemma by either pushing the man with the joystick or by choosing to do nothing. The response time was selected based on that adopted in previous virtual paradigms [12]. As the trolley car approached and at a marked time interval, the people on the tracks began to shout for help. If the large person was pushed, he would also shout. The virtual environment also included other salient features including the sound of the trolley car approaching. After the trolley car had either collided with the large person’s body or with the people on the tracks, participants were left in the virtual environment for a further five seconds to ensure that they had seen and understood the consequences of their actions.

In the judgment condition, participants were given vignettes describing the footbridge dilemma [6] embedded in a further nine distracter moral dilemmas. These moral dilemmas were selected from those originally used in Greene, Sommerville [5] and were presented electronically in a random order. In the final section of each dilemma, participants were asked (“*Is it morally acceptable to [specific to the scenario]*?”). After a response was given, a second action-choice question was displayed asking (“*Would you do it*?”). Participants were given ten seconds to respond to each question matching the response time given in the virtual dilemma. Participants responded by selecting “Yes” (Y) or “No” (N). Responses and response times were recorded.

### Physiological Measures

Heart rate was recorded using a Cateye PL-6000 heart rate monitor in both conditions before and after control and experimental tasks. This provided a means of examining arousal [12, 13]. The ear lobe clip was attached before participants began the electronic questionnaire to ensure that the device was working and to ensure that participants could adjust to the set-up. The rate of heart rate change (bpm) is not stable and can be either gradual or abrupt. As such, heart rate readings were taken at onset and offset of the current task. The duration of time between onset and offset heart rate readings was dependent on task type and for the judgment tasks, determined by self-paced reading speed.

In the action condition, heart rate readings were taken at the onset and offset of the VR control task. The onset was defined as the moment in which the VR task started. Offset was defined as the moment when the VR task automatically stopped after participants had pushed the object. This task was self-paced. Heart rate readings were also taken at the onset of the experimental task (when the VR task started) and at the offset, which was defined as five seconds after the temporal events in the virtual scenario had played out (i.e., the train had collided with the large person or the five people on the tracks). The time between onset heart rate and offset heart rate was 90 seconds. Heart rate sampling was done to assess whether changes in arousal were a result of the moral dilemma itself rather than the novelty of being in VR.

In the judgment condition, heart rate measurements were taken at the onset and offset of the control trial. In the judgment condition, the length of the task was self-paced as a result of individual reading speeds. In the experimental trials, heart rate readings were taken on presentation of the first text section of the footbridge dilemma (onset) and after participants had responded to the dilemma (offset). Heart rate change was subsequently calculated for the control task and for the footbridge dilemma specifically.

### Procedure

All participants first completed the electronic questionnaires before being randomly allocated to one of the two conditions. In the judgment condition, all scenarios were presented on a computer running E-prime software. In the action condition, participants first completed an electronic pre-questionnaire assessing their gaming experience (hours per week of video game play and number of games played annually). For the moral task, a single scenario was presented via the Oculus Rift head-mounted display which was setup using the Oculus SDK 1 development kit. The Oculus Rift is a head mounted VR system that provides an immersive, motion tracked, 3-D experience. The device uses a 7-inch screen with a colour depth of 24 bits per pixel generating a VR environment with a wide field of view (110° diagonal) and resolution of 1280 x 800 pixels (640 x 800 per eye). Head orientation tracking is enabled via a head tracker which runs at 250 Hz. During the task, the participants also wore a pair of Sennheiser headphones and interacted with the scene using a joystick.

## Results

### Pre-questionnaire responses

For the action condition, endorsing a utilitarian outcome and pushing the man in VR was not associated with prior gaming experience (*ps* >.11).

### Moral responses

**Fig 2:**
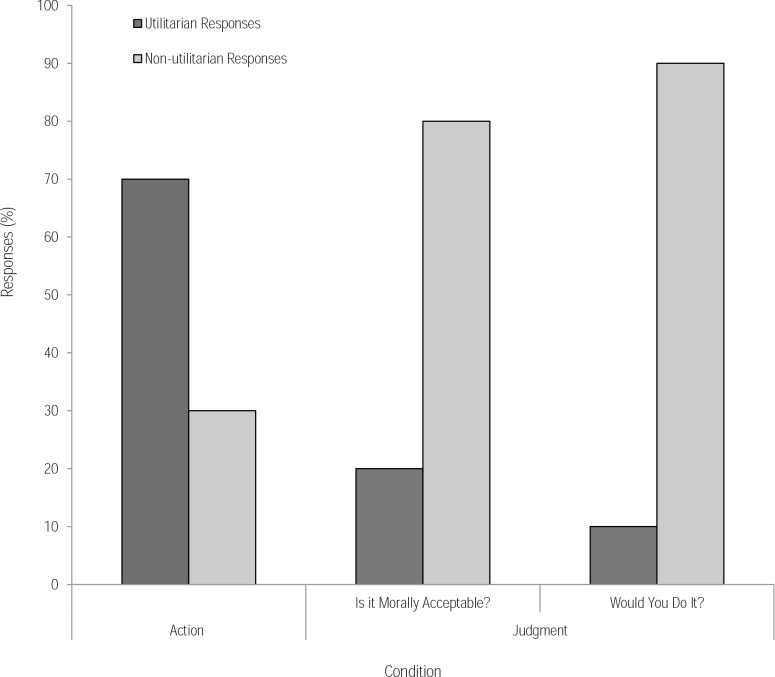
Responses (%) in the action condition and judgment condition in response to the footbridge dilemma. In the judgment condition, participants were asked whether the action was morally acceptable and whether they would do it. A greater number of utilitarian outcomes were endorsed in the action condition

First, we compared responses from the footbridge moral dilemma in the text-based judgment condition with those from the virtual action condition. In the judgment condition, when asked if the action was morally acceptable, 20% of participants endorsed a utilitarian response (i.e., judge that they regard pushing the man as morally acceptable). In the action condition, 70% of participants endorsed a utilitarian response, significantly more than in the judgment condition, (χ^2^(1) = 10.10, *p* = .001). The odds of participants endorsing a utilitarian response were 9.33 times higher in the action condition than in the judgment condition. When asked if they would perform the action (action-choice question) in the judgment condition, 10% of participants endorsed a utilitarian response to the footbridge dilemma, compared to the 70% who endorsed the action in the virtual dilemma, (χ^2^(1) = 15.00, *p* < .001) (Fig 2). This is consistent with Patil et al’s account of Cushman’s theory [12] and its application in VR; visual saliency highlights the negative outcome associated with inaction and this begins to outweigh the negativity associated with the action itself.

In the judgment condition for the text-based version of the footbridge dilemma, no significant difference was found when comparing responses to the judgment question (i.e., moral acceptability) and the action-choice question (i.e., whether they would do it), (*p* = .625).

### Heart rate analyses

**Fig 3:**
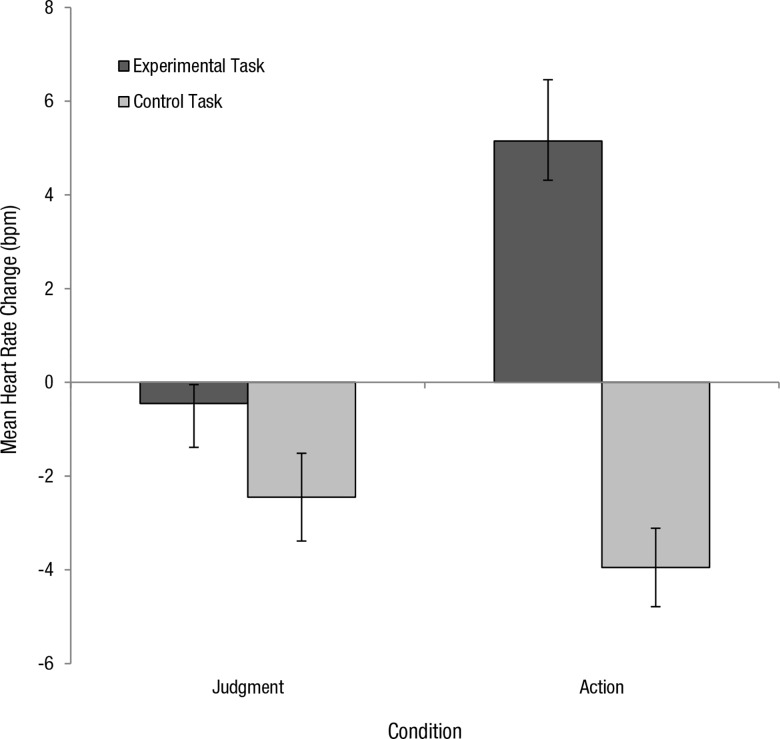
Mean heart rate change (bpm) for control and experimental tasks in both the judgment and action conditions. Increased heart rate was observed in the virtual moral dilemma. Error bars represent +- 1 SE

In both the judgment and action conditions, heart rate change was computed by calculating the difference between heart rate readings (bpm) taken at the onset of the task and heart rate (bpm) taken at the offset of the task. Heart rate changes were computed for each participant in the control task and in the experimental task of their assigned condition. These were averaged to produce mean heart rate change for control and experimental tasks in each condition. In the judgment condition, heart rate decreased during the control task (*M =* -2.45, *SD* = 4.19) and in the experimental task (*M* = -0.45, *SD* = 1.79). In the action condition, heart rate decreased for the control task (*M* = -3.95, *SD* = 3.75) but increased for the experimental task (*M* = 5.15, *SD* = 5.84) (Fig 3).

We conducted a mixed ANOVA with task (control task; experimental task) as within-subjects factor and condition (judgment, action) as the between-subjects factor. Analysis revealed a main effect of task, (*F*(1, 38) = 41.51, *p* = < .001, *η*_*p*_^*2*^ = .52) condition, (*F*(1, 38) = 4.28, *p* = .045, *η*_*p*_^*2*^ = .10) and a significant interaction of task x condition, (*F*(1, 38) = 16.99, *p* = < .001, *η*_*p*_^*2*^ = .31). To further investigate this interaction, simple effects analyses were performed comparing heart rate change in control and experimental tasks in both conditions. In this case, a MANOVA was used to assess simple effects as it results in a smaller error term. This analysis suggested that for the control task, heart rate changes were not significantly different between judgment and action conditions, (*F*(1, 38) = 1.43, *p* = .240). However, for the experimental task, analysis suggested that heart rate changes were significantly different between conditions, (*F*(1, 38) = 16.80, *p* = < .001, *η*_*p*_^*2*^ = .31). Specifically, in the action condition, heart rate changes were significantly greater in the virtual experimental task than in the control task (*F*(1, 38) = 55.80, *p* = < .001, *η*_*p*_^*2*^ = .60). These findings suggest that the modality of VR alone is not responsible for increases in arousal but rather its interaction with moral content.

We assessed whether heart rate change was associated with an increase in utilitarian responses in logistic regression models incorporating heart rate change and its interaction effect with condition (judgment [judgment question, action-choice question], action). As expected, the regression supported previous chi-square analyses (Fig 2) revealing a positive relationship between condition (referencing action condition) and the odds of endorsing a utilitarian response. This was the case when using the judgment question (*b* = 2.23, Wald *X*^*2*^(1) = 9.06, *p* = .003) and also the action-choice question (*b* = 3.05, Wald *X*^*2*^(1) = 11.68, *p* = .001). However, the analysis revealed no significant relationship between heart rate changes and the likelihood of endorsing a utilitarian response in either condition (*ps* >.359). These findings fail to support our secondary hypothesis that increased arousal might predict non-utilitarian responses based on existing dual-process models.

### Personality trait analyses

In order to assess any differences between personality traits across both the judgment and action conditions, a one-way ANOVA was used to compare trait measures. No significant differences between the judgment and action conditions were found (all *ps* > .313), except for Conscientiousness, (*F*(1, 38) = 4.25, *p* = .046, *η*_*p*_^*2*^ = .10) which was higher in the action condition. Emotionality, (*F* (1, 38) = 3.84, *p* = .058) and Openness, (*F*(1, 38) = 3.63, *p* = .064) were marginally significantly different between conditions (see Table 1).

**Table 1:** Means and standard deviations for LPS, HEXACO-PI-IR and IRI subscales.

Measure	Subscale	Condition	
		Judgment	Action
		M*(SD)*	M*(SD)*
1. LPS			
	Primary	28.70*(6*.*62)*	28.70*(5*.*36)*
	Secondary	21.60*(4*.*52)*	19.70*(4*.*03)*
2. HEXACO			
	H	3.58*(0*.*73)*	3.53*(0*.*60)*
	Em	3.32*(0*.*70)*	3.74*(0*.*67)*
	Ex	3.23*(0*.*50)*	3.37*(0*.*51)*
	A	3.11*(0*.*78)*	3.03*(0*.*65)*
	C	3.47*(0*.70)*	3.86*(0*.47)*
	O	3.53*(0*.*72)*	3.11*(0*.*67)*
3. IRI			
	PT	19.45*(5*.*29)*	18.10*(5*.*10)*
	EC	17.80*(5*.*86)*	19.15*(5*.*63)*
	PD	11.35*(5*.*11)*	12.45*(6*.*39)*
	FS	20.40*(6*.*04)*	21.35*(3*.*95)*

*Note*: H = Honesty-Humility, Em = Emotionality, Ex = Extraversion, A = Agreeableness, C = Conscientiousness, O = Openness to experience. PT = Perspective Taking, EC = Empathic Concern, PD = Personal Distress, FS = Fantasy Seeking.

*p < .05

First, in order to determine whether psychopathic traits predicted utilitarian responses as in previous studies (e.g., [20]), we conducted univariate logistic regressions with condition (judgment, action) as the selection variable, primary and secondary Psychopathy as the continuous predictors and response as the categorical outcome (non-utilitarian, utilitarian). In the judgment condition, we analysed responses to both action-choice and judgment questions. For the judgment condition, neither dimension of Psychopathy was a significant predictor of utilitarian responses to each question (*ps* >.159). In the action condition, primary Psychopathy was a marginally significant predictor of utilitarian responses, (*b* = 0.21, Wald *X*^*2*^(1) = 3.54, *p* = .060) (see Table 2). This finding is consistent with previous findings and our hypothesis that anti-social traits predict utilitarian responses (e.g., 20, 23). However, we fail to support the distinction between action-choice and judgment found in previous research [20].

**Table 2:** Logistic regression with primary Psychopathy as predictor.

			95% CI for Odds Ratio		
		B (*SE*)	Lower	Odds Ratio	Upper
**Included**					
**Constant**		-4.98 (*3*.*09*)			
**Psychopathy**					
	**Primary**	0.21* (*0*.*11*)	0.99	1.23	1.53

*Note*: *R*^*2*^ = .18 (Hosmer & Lemeshow), .20 (Cox & Snell), .28 (Nagelkerke). *Model X*^*2*^(1) = 4.38, *p* = .04.

*p = .06. (*SE)* = standard error.

Honesty-Humility, a trait negatively correlated with primary Psychopathy (*r*(40) = -.73, *p* < .001), was found to be a significant negative predictor of utilitarian responses in a second univariate logistic regression (including all HEXACO traits) in the action condition, (*b* = -2.53, Wald *X*^*2*^(1) = 3.95, *p* = .047) (see Table 3). For the judgment condition, Honesty-Humility was not a significant negative predictor of utilitarian responses (*ps* >.256).

**Table 3:** Logistic regression with Honesty-Humility as predictor.

			95% CI for Odds Ratio		
		B (*SE*)	Lower	Odds Ratio	Upper
Included					
Constant		-2.53 (*1*.*27*)			
HEXACO					
	H	1.19* (*0*.*67*)	0.07	0.08	0.97

*Note*: H = Honesty-Humility. *R*^*2*^ = .25 (Hosmer & Lemeshow), .26 (Cox & Snell), .37 (Nagelkerke). *Model X*^*2*^(1) = 6.01, *p* = .01.

*p = .05. (*SE)* = standard error.

The four components of empathy were not found to be significant predictors of response type in the judgment condition (*ps* = .271) or the action condition (*ps* = .073).

## Summary and Discussion

Study 1 found that participants endorsed the utilitarian response of pushing when action was required in VR, but refused to endorse the same response when judgment was required in the text-based counterpart. Heart rate change was primarily assessed to determine whether arousal was triggered by modality or moral content. In the present study, heart rate change was highest in the action condition when participants completed the virtual moral task while there was no difference between groups in arousal in the control tasks. This suggests that the VR modality alone was not responsible for this increased arousal. Subsequent analysis found that heart rate change did not predict moral responses in either condition. However it is important to note that arousal responses in the present action paradigm were assessed not only across the moment of decision-making as in previous research [13] but also during the time in which participants witnessed the consequences of their actions (or omissions of action). This may explain why arousal did not predict moral responses in the present study. Additionally, previous gaming experience did not predict utilitarian responses in the action condition; this might suggest that responses in the virtual moral dilemma were not akin to those of a gaming environment.

As a secondary finding, primary Psychopathy was found to be a marginal predictor of the endorsement of virtual action responses. Honesty-Humility was found to be a negative predictor of this endorsement in the action condition only. This can be explained given the association between Honesty-Humility and traits such as fairness and sincerity. These traits are contrasted with those associated with the Dark Triad of personality (Psychopathy, Narcissism and Machiavellianism) [29] giving it an inverse association with Psychopathy (e.g., [23]). Although we found that empathy was not a significant predictor in either condition, it is likely that this was due to its measurement of both cognitive and emotional empathy; cognitive empathy remains intact in those individuals who display psychopathic traits [30].

In the present methodology, it could be argued that the incorporation of a joystick device in the virtual moral dilemma compared to key-based responses in the text-based dilemmas resulted in game-related affordance effects; the joystick itself may have primed pushing responses. In order to inspect this further, a short follow-up study was carried out (Study 1.1). Additionally, the nature of the sample in the current study constrains the reliability of these results. The present sample was limited in several ways comprising undergraduate psychology students (*M =* 26.00 years old, *SD* = 9.77 years, 35 females). Thus in Study 2, we aimed to replicate the existing methodology but with a qualitatively different sample.

## Study 1.1

This follow-up study was designed to address possible influences of the inclusion of a joystick device in the virtual personal moral dilemma described in Study 1.

## Method

### Participants

Forty participants (74.4% female, 25.6% male, *M*_*age*_ *=* 22.37, *SD* = 6.79) were recruited from the Plymouth University, School of Psychology, participant pool and participated for course credit.

### Moral judgment measures

The study comprised two conditions to which participants were randomly allocated; a joystick-response condition (*N* = 20) and a key-response condition (*N* = 20). In both conditions, participants were presented with ten personal text-based moral dilemmas taken from an existing database including the footbridge dilemma [6]. In the final section of each dilemma, participants were asked (“*Is it morally acceptable to [specific to the scenario]*?”) followed by an action-choice question (“*Would you do it*?”). Participants were given ten seconds to respond to each question. In the key-response condition, participants responded using the key-responses described in the judgment condition of the present research; by selecting “Yes” (Y) or “No” (N). In the joystick-response condition, participants responded using a joystick; pushing forward to elicit a “Yes” response and clicking a side button to elicit a “No” response. This setup allowed a direct assessment of the potential affordance effects triggered by the inclusion of a joystick device.

### Procedure

In both conditions, moral dilemmas were presented in a random order on a computer running e-prime software and participants responded using either the keyboard or joystick based on condition assignment.

## Results

### Moral responses

The proportion of utilitarian responses was calculated for each participant across question type. A mixed ANOVA with question type (judgment, action-choice) as within-subjects factor and condition (key-response, joystick-response) as the between-subjects factor revealed no significant differences between condition (*p =* .923), question (*p =* .472) and no interaction effect (*p* = .295).

## Summary and Discussion

This follow-up study found no significant effect of response device on moral decision-making. As such, we found no reason to alter the joystick response option from the present virtual moral dilemma; the increase in utilitarian responses was not likely induced by game-related affordance effects.

## Study 2

In Study 1, convenience sampling of undergraduate students created a narrow database subsequently limiting the replicability of our findings. Study 2 was designed to address these sample limitations. We examined the same research hypotheses as in Study 1 leaving all methodological procedures identical. The personality variables included in this study were limited to primary Psychopathy (16 items; α = .72), secondary Psychopathy (10 items; α = .43) and Honesty-Humility (10 items; α = .76), as these were found to be associated with moral responses in Study 1.

## Method

### Participants

Sixty two participants comprising 41 females and 21 males (*M*_age_ *=* 31.10 years old, SD = 15.54 years, *18 to 71 years*) were recruited from the public in Plymouth, Devon (UK) and the surrounding area using online advertisements. Participants were paid for their participation in the study. All participants had normal or corrected-to-normal vision. The majority of participants were right-handed (81.7%). Two participants were excluded from the study as they failed to complete the task due to lack of understanding. As such, 60 participants comprising 41 females and 19 males (*M*_age_ *=* 30.05, SD = 14.55 years, *18 to 68 years*) comprised the final sample. As in Study 1, participants were randomly allocated to a judgment condition (*N* = 30) or an action condition (*N* = 30).

## Results

### Pre-questionnaire responses

For the action condition, endorsing a utilitarian outcome and pushing the man in VR was not associated with prior gaming experience (*ps* >.31).

### Moral responses

**Fig 4:**
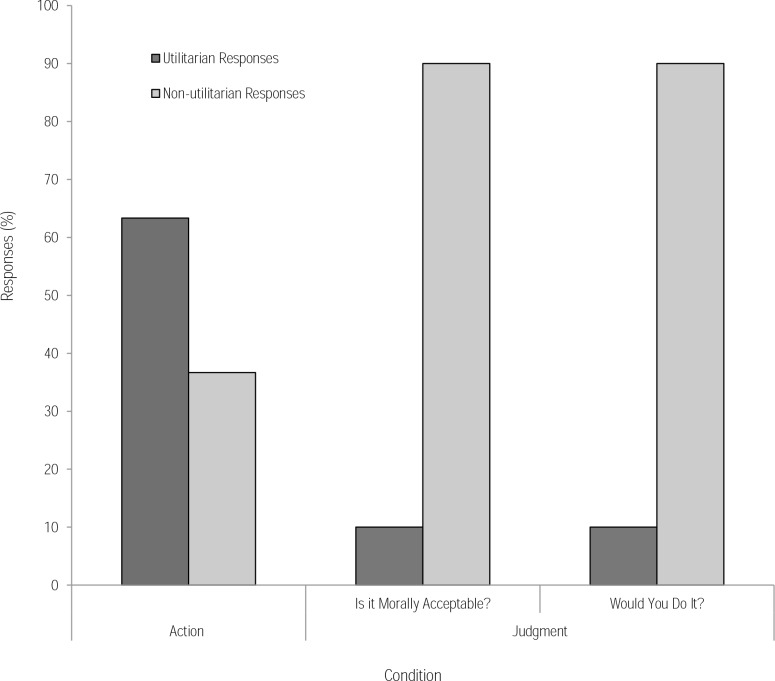
Responses (%) in the action condition and judgment condition in response to the footbridge dilemma in Study 2. In the judgment condition, participants were asked whether the action was morally acceptable and whether they would do it. A greater number of utilitarian outcomes were endorsed in the action condition.

First, we compared responses from the footbridge moral dilemma in the theoretical judgment condition with those from the virtual action condition. In the judgment condition, when asked if the action was morally acceptable, 10% of participants endorsed a utilitarian response (i.e., judge that they regard pushing the man as morally acceptable). In the VR action condition, 63.3% of participants endorsed a utilitarian response, significantly more than in the judgment condition, (χ^*2*^(1) = 18.37, *p* < .001). The odds of participants endorsing a utilitarian response were 15.55 times higher in the action condition than in the judgment condition. When asked if they would perform the action (action-choice question) in the judgment condition, the same responses were observed with 10% of participants endorsing a utilitarian response to the hypothetical footbridge dilemma, compared to the 63.3% who endorsed the action in the virtual dilemma, (χ^*2*^(1) = 18.37, *p* < .001) (Fig 4). As in Study 1, this finding is consistent with our hypothesis based on Patil et al’s research [12]; the visual saliency in VR emphasises the negative outcome in the moral dilemma and this begins to outweigh the negative value assigned to the action of pushing.

In the judgment condition for the text-based version of the footbridge dilemma, no significant difference was found when comparing responses to the judgment question (i.e., moral acceptability) and the action-choice question (i.e., whether they would do it), (*p* = 1.00).

### Heart rate analyses

**Fig 5:**
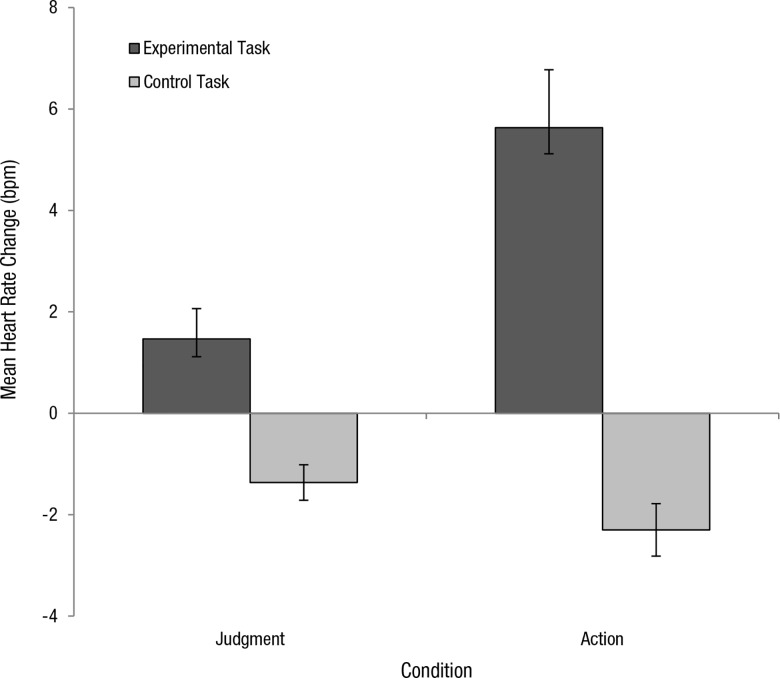
Mean heart rate change (bpm) for control and experimental tasks in both the judgment and action conditions. Heart rate change was significantly higher in the virtual moral dilemma. Error bars represent +- 1 SE

In the judgment condition, heart rate decreased during the control task (*M =* -1.37, *SD* = 1.92) and increased in the experimental task (*M* = 1.47, *SD* = 3.27). In the action condition, heart rate decreased for the control task (*M* = -2.30, *SD* = 2.83) and also increased for the experimental task (*M* = 5.63, *SD* = 6.25) (Fig 5).

We conducted a mixed ANOVA with task (control task; experimental task) as within-subjects factor and condition (judgment, action) as the between-subjects factor. Analysis revealed a main effect of task, (*F*(1, 58) = 57.50, *p* = < .001, *η*_*p*_^*2*^ = .50) condition, (*F*(1, 58) = 5.03, *p* = .029, *η*_*p*_^*2*^ = .08) and a significant interaction of task x condition, (*F*(1, 58) = 12.90, *p* = .001, *η*_*p*_^*2*^ = .18). To further investigate this interaction, a MANOVA was carried out to assess simple effects, comparing heart rate change in control and experimental tasks in both conditions. Analysis suggested that heart rate changes were significantly greater in the experimental tasks than in the control tasks in both the action condition (*F*(1, 58) = 62.43, *p* = < .001, *η*_*p*_^*2*^ = .52) and the judgment condition (*F*(1, 58) = 7.96, *p* = .007, *η*_*p*_^*2*^ = .12). However, for the experimental task, heart rate changes were significantly higher in the action condition compared to the judgment condition, (*F*(1, 58) = 10.47, *p* = 002, *η*_*p*_^*2*^ = .15). For the control task, heart rate changes were not significantly different between the judgment and action conditions, (*F*(1, 58) = 2.23, *p* = .140). As in Study 1, these findings suggest that increases in arousal are triggered by the interaction between modality and moral content.

As in Study 1, we assessed whether heart rate change was associated with an increase in utilitarian responses in logistic regression models. As expected, the regression supported previous chi-square analyses (Fig 4) revealing a positive relationship between condition (referencing action condition) and the odds of endorsing a utilitarian response. This was the case when referencing the judgment condition using the judgment question (*b* = 2.74, Wald *X*^*2*^(1) = 14.65, *p* < .001) and also the action-choice question (*b* = 2.74, Wald *X*^*2*^(1) = 14.65, *p* = .008). However, the analysis revealed no significant relationship between heart rate changes and the likelihood of endorsing a utilitarian response in either condition (*ps* >.088). Again, these findings fail to support our secondary hypothesis that increased arousal might predict non-utilitarian responses based on existing dual-process models.

### Personality trait analyses

In order to assess any differences between personality traits across both the judgment and action conditions, a one-way ANOVA was used to compare trait measures. A significant difference was found in both primary Psychopathy (*F*(1, 58) = 22.09, *p* < .001, *η*_*p*_^*2*^ = .28) and secondary Psychopathy *F*(1, 58) = 7.55, *p* = .008, *η*_*p*_^*2*^ = .12) between the action and judgment conditions; higher primary Psychopathy (*M* = 37.07, *SD* = 3.75) and secondary Psychopathy scores (*M* = 21.97, *SD* = 2.94) were observed in the action condition compared to the judgment condition primary Psychopathy score (*M* = 30.60, *SD* = 6.54) and secondary Psychopathy score (*M* = 19.67, *SD* = 3.52). No differences were found between Honesty-Humility scores (*p* = .529) (see Table 4).

**Table 4:** Means and standard deviations for Psychopathy subscales and Honesty-Humility.

Measure	Subscale	Condition	
		Judgment	Action
		M*(SD)*	M*(SD)*
2. LPS			
	Primary	30.60*(6*.54)**	37.07*(3*.75)**
	Secondary	19.67*(3*.52)*	21.97*(2*.94)*
2. HEXACO			
	H	3.40*(0*.*60)*	3.50*(0*.*62)*

*Note*: H = Honesty-Humility.

*p < .05

** *p* < .001

As in Study 1, we conducted univariate logistic regressions with condition (judgment, action) as the selection variable, Psychopathy subscales as the continuous predictors and response as the categorical outcome (non-utilitarian, utilitarian). In the judgment condition, we analysed responses to both action-choice and judgment questions. Psychopathy subscales were not significant predictors of utilitarian responses in either the judgment condition for both questions (*ps* >.802) or in the action condition (*ps* >.207).

Additionally, Honesty-Humility was not a significant negative predictor of utilitarian responses in either the judgment condition for both questions (*ps* >.601) or in the action condition (*p* = .787). Contrasting with Study 1, these findings fail to support our hypothesis that antisocial traits and associated traits predict moral responses.

## Summary and Discussion

Study 2 added support for the finding that participants endorse the utilitarian response of pushing when action is required in VR but refuse to endorse the same response when judgment is required in hypothetical text-based dilemmas. As in Study 1, heart rate significantly increased for the virtual moral task in the action condition whereas no differences were found between conditions in arousal in control tasks. As before, this indicates that the VR modality alone was not responsible for increased levels of arousal. Subsequent analysis found that arousal did not predict moral responses. As in Study 1, this may have been due to the heart rate sampling period which incorporated the time in which participants witnessed the consequences of their actions. Again, previous gaming experience did not predict utilitarian responses in the action condition. Contrary to Study 1, Psychopathy and Honesty-Humility were not found to predict moral actions in VR.

## Discussion

The trolley problem has long offered moral philosophers and psychologists a way of comparing utilitarian and deontological philosophies “in one neat little puzzle” ([31], p. 116). These text-based dilemmas, whilst being ideal in their experimental simplicity, raise questions about peripheral contextual features and their influence on moral decision-making [12]. Whilst research has already addressed this shortcoming in virtual reconstructions of impersonal moral dilemmas [12–14], these studies are the first to incorporate a virtual personal moral dilemma.

### Behavioural Responses

Overall, we found that participants behaved differently in judgment-based formulations and action-based virtual formulations of the same moral dilemma. In both Studies 1 and 2, participants in the action condition, who responded to a virtual personal dilemma, endorsed a greater proportion of utilitarian responses than those who responded to the same text-based dilemma.

At this early stage, our results appear to fall in line with the theory of Cushman regarding outcome and action-based value representations [7]. Given the contextual saliency of the virtual footbridge dilemma, outcome-based value representations for not pushing the man and allowing the people on the tracks to be killed, might have had a greater negative value [7]. This may have been greater than the action-based negative value representation for pushing the man to his death. Indeed, in Patil, Cogoni [12], the authors propose a similar theory in which there may have been greater outcome-based value representations for not acting and allowing individuals to be harmed, leading to a greater number of utilitarian responses. As adapted from Patil, Cogoni [12], we suggest that the saliency in the present studies may have been generated by the personal nature of the dilemma and the ability to see potential victims on the tracks. In the text-based dilemmas, the absence of salient features and reliance on imagination might have led to assignment of negative value to the action of harming as opposed to the outcome of not acting [7]. This theory is tentative and requires further investigation. Future research might consider incorporating eye-tracking software within virtual headset devices to measure gaze durations for victims and non-victims; arguably this could indicate for which person(s) there is greatest concern, although findings in this area are mixed [14, 32].

### Emotional Arousal

Heart rate change was monitored in order to determine whether increases in emotional arousal would be triggered by modality or moral content. In Study 2, both the moral text-based dilemma and virtual moral task were more arousing than their control counterparts with the virtual moral task eliciting the greatest increase in arousal overall. In Study 1, the virtual moral task was more arousing than the control counterpart and also the moral text-based dilemma. This suggests that the modality of VR alone was not responsible for changes in heart rate but rather the moral nature of the virtual task.

Given previous theories regarding the association between non-utilitarian responses and emotional arousal in personal moral dilemmas [5], we might expect an increase in heart rate to predict non-utilitarian responses. Previous virtual studies have measured emotional arousal and have yielded mixed findings; whilst Navarrete, McDonald [13] found that autonomic arousal was negatively related to utilitarian responses in VR, Patil, Cogoni [12] found that arousal was highest in virtual moral dilemmas and this corresponded with a greater proportion of utilitarian responses. In the present research, we also found an overall increase in emotional arousal in the action conditions for the virtual moral dilemma but in both Study 1 and Study 2, we did not find that heart rate changes directly predicted moral responses in either condition.

It is important to note here that heart rate change in the action condition also incorporated the moment in which participants witnessed the consequences of their actions and as such recorded arousal beyond the decision-making process itself. The main purpose of arousal assessment in the present research was to examine whether changes in arousal were as a result of modality or moral content. Whilst the finding that arousal was greatest in the virtual moral task suggests that modality alone is not responsible for this increase, it is important to acknowledge an additional explanation; witnessing consequences in the action condition may have led to greater emotional arousal compared to imagining consequences in the judgment condition. As a result of this and given that this research is the first to implement a personal moral dilemma in VR, implications of these results for Greene’s dual process model remain unclear.

### Personality Traits

In Study 1, we found that psychopathic traits marginally predicted and Honesty-Humility negatively predicted utilitarian endorsements in the action condition in VR but not in the text-based judgment condition. This might fall in line with previous research finding that psychopaths have distinct moral judgments and actions [21, 22]. More generally, it might give support to the viewpoint that moral judgment and moral action remain dissociated. However, these conclusions are given tentatively as Study 2 failed to support these findings. Importantly, in Study 2, differences in Psychopathy scores prevented a robust follow-up of the findings from Study 1. As such, further research examining the role of pro- and anti-social traits in these action frameworks is required prior to further interpretation.

### Alternative Interpretations

Whilst Cushman’s action, outcome and value model seems to offer a convincing interpretation of these studies’ findings, there are alternative explanations in need of addressing.

Firstly, the potential interpretation that the increase in virtual utilitarian responses could be a result of artificial gaming behaviours as opposed to moral-based decision making is not supported in the present research. We found that decisions made in the virtual moral dilemma were not influenced by previous gaming experience; as such, video game desensitization cannot explain these findings. Additionally, we found no game-related affordance effects as a result of the incorporation of a joystick device in Study 1.1 and as such, we argue that the increased utilitarian response pattern found in VR was not likely induced by this.

In terms of further differences between the modalities of the virtual moral dilemma and its text-based counterpart, whilst we attempted to match the temporal nature of the paradigms, it might be argued that salient auditory cues in the virtual dilemma resulted in the present outcome. Specifically, in the virtual dilemma, the victims on the track began to yell at a fixed time interval during the dilemma whilst the man on the bridge did not yell until pushed; potentially leading to the victims calling attention to themselves, whilst the man remained mute until after the participant had responded. In an attempt to investigate this, we compared those individuals who gave a utilitarian response prior to hearing the victims yell, to those who did after hearing the victims yell. In Study 1, of the 70% of individuals that endorsed a utilitarian response, 7.1% executed the action after the victims had begun to yell. In Study 2, of the 63.3% utilitarian responses, 16.6% elicited this response after hearing the victims yell. This indicates that in both studies, the majority of participants in VR chose to push before the victims had called attention to themselves. As such, the response patterns found in the virtual moral dilemma were not likely triggered by these salient auditory cues.

Differences in auditory cues, with regards to instructions, must also be considered. In the judgment condition, the text-based vignette explicitly draws equal attention to the acts of killing and saving; “the stranger will die if you do this, but the five workmen will be saved” [10]. In the action condition however, the verbal instructions explicitly refer to saving; “…if you want to save the people, you could push the large man…”. As such, it could be argued that the lack of conflict created in the virtual scenario biased participants’ attention to the act of saving. Despite this, we would argue that the visual saliency of the virtual scenario makes the act of killing the man explicit; there is no reliance on imagination in the virtual scenario and the consequences of your action (or omission of action) can be processed visually. Future research should explore this potential bias further.

Importantly, with regards to the general criticisms of the ‘unreality’ of VR, previous research has found that VR offers a platform in which sensitive topics can be studied in a more ecologically valid way; with individuals responding realistically in virtual paradigms (e.g., [33]). Whilst we do not aim to predict real-life behaviours in these hypothetical moral dilemmas, whether virtual or not, this action framework could offer insights into the immediate responses experienced in emotionally aversive situations.

Finally, it might be that an alternative explanation for these studies’ findings rests in an embodied-cognition perspective of moral decision-making. The significance of this field to moral emotion research has been extensive. For example, research has shown that the physical experience of cleanliness [34] and feelings of disgust [35] can prime harsher moral judgments. Importantly, this phenomenon appears to extend to concepts such as importance, with greater physical exertion resulting in greater judgments of importance for example [36]. In this case, perception of the physical consequences of action may have elicited learned experiences and subsequently resulted in greater importance being assigned to action than when facing a more abstract text-based dilemma. This embodied perspective should be investigated within future virtual paradigms. This might be done by incorporating haptic feedback with varying resistance or weight to see how moral decision-making is subsequently affected.

### Methodological Considerations

Although we addressed the problem of excluding personal dilemmas in VR research [12], we acknowledge the limitation of including a single virtual reconstruction of the footbridge dilemma in the present research; as such, the generalizability of our results is limited in the broader moral decision-making literature. However, given that previous virtual paradigms have considered only impersonal moral dilemmas, this research has offered initial insights into the immediate emotional responses prompted by a novel simulation of a personal moral dilemma. Future research might consider constructing multiple personal moral dilemmas in VR in order to investigate further personal factors such as physical contact and spatial proximity in this action framework.

Additionally, given the increase in use of virtual simulation training paradigms across emergency and healthcare services as a means of assessing emotionally conflicting decision-making processes, future research should consider expanding the present virtual paradigms to include life-like scenarios, extending the generalization of findings concerning moral actions.

Despite these shortcomings, this research has provided initial headway in advancing the moral domain beyond self-report assessments into behavioural decision paradigms.

### Conclusions

In summary, this research has demonstrated dissociation between moral actions endorsed in a virtual framework, and moral judgments endorsed in a traditional format. Importantly, although in its initial stages, this VR technology has allowed us to investigate personal moral dilemmas with greater ecological validity than traditional paradigms. This has opened opportunities for the application of VR methods in social psychology, allowing the examination of sensitive issues in a way not previously possible.
