# Correction: Virtual Morality: Transitioning from Moral Judgment to Moral Action?

**DOI:** 10.1371/journal.pone.0170133

**Published:** 2017-01-11

**Authors:** Kathryn B. Francis, Charles Howard, Ian S. Howard, Michaela Gummerum, Giorgio Ganis, Grace Anderson, Sylvia Terbeck

There is an error in Table 3. The beta coefficients (B) for the constant and trait are incorrectly swapped. The authors have provided a corrected table here.

**Table 3:** Logistic regression with Honesty-Humility as predictor

			95% CI for Odds Ratio		
		B (*SE*)	Lower	Odds Ratio	Upper
Included					
Constant		1.19 (*0*.*67*)			
HEXACO					
	H	-2.53* (*1*.*27*)	0.07	0.08	0.97

*Note*: H = Honesty-Humility. *R^2^* = .25 (Hosmer & Lemeshow), .26 (Cox & Snell), .37 (Nagelkerke). *Model X^2^*(1) = 6.01, *p* = .01.

*p = .05. (*SE)* = standard error.
